# Persistent Nipple Pain in Breastfeeding Mothers Associated with Abnormal Infant Tongue Movement

**DOI:** 10.3390/ijerph120910833

**Published:** 2015-09-02

**Authors:** Holly L. McClellan, Jacqueline C. Kent, Anna R. Hepworth, Peter E. Hartmann, Donna T. Geddes

**Affiliations:** School of Chemistry & Biochemistry, The University of Western Australia, Crawley, WA 6009, Australia; E-Mails: mcclellan.holly@gmail.com (H.L.M.); Anna.Hepworth@uwa.edu.au (A.R.H.); Peter.Hartmann@uwa.edu.au (P.E.H.); Donna.Geddes@uwa.edu.au (D.T.G.)

**Keywords:** breastfeeding, infant, lactation, nipple pain, sucking behaviour

## Abstract

*Background*: Infants of breastfeeding mothers with persistent nipple pain have been shown to apply stronger vacuums to the breast and transfer less milk during one monitored feed. This may be associated with differences in the movement of the tongue. The aim was to analyse the intra-oral nipple shape and movement of the tongue of infants of mothers with and without nipple pain. *Methods*: Breastfeeding infants of mothers with or without nipple pain were monitored using ultrasound and intra-oral vacuum during one breastfeed. From cine clips of the ultrasound scans measurements were made of the depth of the intra-oral space between the hard-soft palate junction (HSPJ) and the mid-tongue; the distance of the tip of the nipple to the HSPJ; and nipple diameters from the tip to the base. *Results*: During nutritive sucking, tongue movements of infants of mothers with nipple pain resulted in a smaller intra-oral space (*p* = 0.040) and restricted nipple expansion compared to controls (*p* < 0.012). Stronger baseline and peak vacuums compared to controls were confirmed (*p* = 0.002). *Conclusion*: In these mothers, nipple pain was associated with restricted infant tongue movement. Ultrasound may complement measurement of intra-oral vacuum in monitoring treatment strategies in breastfeeding women experiencing nipple pain.

## 1. Introduction

Breast milk is a highly bioactive secretion, with protective, nutritive and developmental functions that are proposed to have evolved from the innate immune system [1] to provide species- and stage-specific protection and nutrition for the infant [2]. Therefore, exclusive breastfeeding until six months of age and continued breastfeeding until two years is recommended to reduce infant mortality and illness [3].

Despite high rates of initiation of breastfeeding, many women introduce supplementary feeds or wean their infants in the first month postpartum due to breastfeeding difficulties such as nipple pain [4,5]. The major cause of nipple pain is considered to be incorrect positioning and attachment of the infant to the breast resulting in strong vacuums [6,7] and it is assumed that correct positioning and attachment can be achieved by all dyads. However, current clinical guidelines are based on experience and adjustment of positioning and attachment is not always successful in alleviating nipple pain. Moreover, the outcome is not monitored objectively, which may be why nipple pain remains a major cause of early weaning.

The application of vacuum to the breast and nipple during breastfeeding has been shown to be important for attachment to the breast, proper positioning of the nipple, and for milk removal [8,9]. Recently, we found that when intra-oral vacuums were measured, infants of mothers with persistent nipple pain applied significantly stronger baseline vacuum (maximum pressure) and peak vacuum (minimum pressure) to the breast and transferred less milk during one monitored feed, despite professional lactation counselling [10]. 

Submental mid-sagittal ultrasound has been used recently to visualise the infant’s intra-oral cavity during breastfeeding. The mid-sagittal plane provides an optimal view of the tongue with the hard-soft palate junction (HSPJ) providing a reliable reference point for performing measurements. Acoustic shadowing from the mandible makes images in the coronal (transbuccal) plane difficult to obtain. Moreover, both the coronal and transverse planes do not display a static landmark that can be utilised either to determine whether the scanning plane is accurate or to make measurements [11,12]. Using submental mid-sagittal ultrasound with simultaneous vacuum measurement during breastfeeding showed that milk flow from the nipple occurred as the tongue lowered from the hard palate, the nipple (which remains in apposition with the anterior tongue [12]) expanded, and the vacuum levels increased [8].

Intra-oral nipple diameter measurements provide a measure of the tongue movement relative to the hard palate during sucking [12]. During normal breastfeeding, as the tongue lowered and vacuum became stronger the nipple diameter increased significantly along the entire length of the imaged nipple [12]. Given the association between tongue movement and the application of vacuum it is possible that strong vacuums measured in the oral cavity of infants of mothers with nipple pain are associated with abnormal tongue movements.

The main focus of this study was the use of submental mid-sagittal ultrasound imaging of the infant’s oral cavity during breastfeeding to make a more detailed analysis of the movement of the tongue and the resulting intra-oral nipple shape, and identify whether there are differences between infants of mothers with and without nipple pain.

## 2. Methods

### 2.1. Subjects

Two groups of breastfeeding mothers of singleton infants were recruited for this study: those who reported persistent nipple pain during breastfeeding (Pain group) and those who did not (Control group), as described previously [10]. The Pain group was recruited by recommendation from an International Board Certified Lactation Consultant if assessment and counselling did not resolve the mother’s pain. Exclusion criteria for the Pain group were maternal nipple infection, vasospasm or dermatitis, or infant ankyloglossia or torticollis. The Control group was recruited through the West Australian Branch of the Australian Breastfeeding Association and local Child Health Nurses. Exclusion criteria for the control group were low milk production, premature delivery, and illness of either the mother or infant.

All participants attended The University of Western Australia research laboratory at the Breast Feeding Centre of King Edward Memorial Hospital. Infants were monitored for an entire breastfeed, with the mothers seated comfortably using the cradle hold, as described previously [8]. Participants supplied written, informed consent to participate in the study, which was approved by the Human Research Ethics Committee of The University of Western Australia.

### 2.2. Ultrasound Imaging

Submental mid-sagittal scans of the infant’s oral cavity were performed for the entire breastfeed. The infants were scanned using a Toshiba SSA-770A/80, Aplio 80 (Tokyo, Japan) with a PVT-661VT transducer and Parker Ultrasonic Gel (Fairfield, NJ, USA). Images were oriented so that the nipple was always on the left side and at the top of the image. Average setting values were gain: 55 db, dynamic range: 60 db, frequency: 8.8 MHz. Two focal zones were used to narrow the ultrasound beam and improve image resolution. One was placed at the hard palate and the other at the nipple–tongue border. All scans were recorded for later analysis.

### 2.3. Ultrasound Measurements of Tongue Action

**Figure 1:**
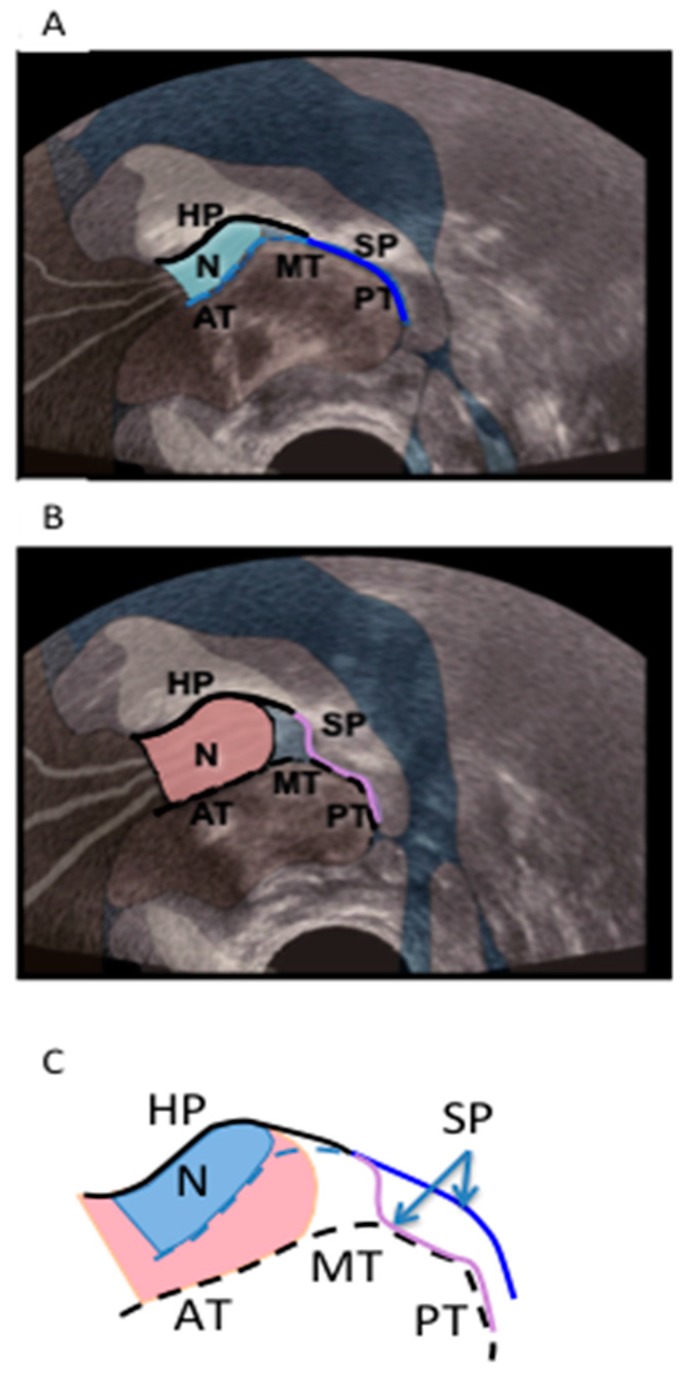
Submental ultrasound image of nipple in infant’s mouth during breastfeeding at, (**A)** the tongue up position, and (**B)** the tongue down position. (**C)** is a diagrammatic representation of the nipple shape and tongue position at tongue up (blue) and tongue down (pink); HP, hard palate; SP, soft palate; N, nipple; AT, anterior tongue; MT, mid-tongue; PT, posterior tongue.

For each infant, the first recorded cine clip of optimal imaging during nutritive sucking that included at least three full suck cycles was isolated from Chart v4.5 (ADInstruments, Castle Hill, NSW, Australia) and stored on a MacBook Pro (Max OSX, V 10.5.7). For each infant, two images were then selected from each of three suck cycles: one when the mid-tongue was raised to its most superior level (tongue up, Figure 1A), and the second when the mid-tongue was lowered to its most inferior level (tongue down, Figure 1B). The following measurements were made on each selected image using Screen Calipers, V. 3.2 (Iconico Inc, New York, NY, USA) as previously described [12]: the depth of the space between the HSPJ and the mid-tongue (intra-oral space depth); the N-HSPJ distance; and nipple diameters at 2, 5, 10 and 15 mm from the nipple tip (location).

### 2.4. Milk Transfer

The amount of milk the infant removed from the breast was measured by test weighing the infant on electronic scales (BabyWeigh Scale, Medela Inc, McHenry, IL, USA) before and immediately after the feed [13]. The amount of milk consumed was calculated as the difference in grams between the final weight and the initial weight. Milk transfer (g/min) was calculated as the amount of milk consumed during the feed (g) divided by the duration of the feed (min). The nutritive portion of the feed was defined as the time during which there was discernable milk flow when the infant was sucking. Nutritive milk transfer (g/min) was calculated as the amount of milk consumed during the feed (g) divided by the duration of the nutritive portion of the feed (min).

### 2.5. Intra-Oral Vacuum Measurement

Intra-oral vacuum (relative to atmospheric, mmHg) was measured via a small Silastic tube (SNS, Medela AG, Baar, Switzerland) filled with sterile water and attached to a pressure transducer (Cobe Laboratories, Frenchs Forest, NSW, Australia) taped alongside and terminating just past the nipple tip. The pressure transducer was connected to a bridge amp (ADInstruments, Castle Hill, NSW, Australia) via an interconnect cable (Cobe Laboratories). The output of the pressure transducer and ultrasound images were channeled to a Power Lab (ADInstruments, Castle Hill, NSW, Australia) and simultaneous recordings were made using the software package Chart v4.5 on a laptop computer(Mac OSX v10.3.8).

### 2.6. Data Analysis

All analyses were performed using R 2.9.0 for Mac OSX [14] using the base packages, and the libraries nlme [15] and multcomp [16], which were used for linear mixed modelling and multiple comparison of means, respectively. Summary statistics are presented as mean ± SD where the Shapiro-Wilk test indicated normality, or median (interquartile range) otherwise, and model parameters are presented as group mean ± SE. *p* < 0.05 was considered to be statistically significant. Missing data was dealt with via available case analysis.

Groups were compared on demographic, milk intake, and vacuum variables using two-tailed independent samples Student’s *t*-test where the Shapiro-Wilk test indicated normality, and Kruskal-Wallis rank sum test otherwise. Categorical variables were compared using Fisher’s exact test. All other analyses used linear mixed models to account for the related nature of the data. Random effects were intercept only, grouped by infant.

Final models were determined using backwards stepwise selection; appropriateness of each subsequent model was tested using ANOVA, with regard to the marginal significance of the highest order interactions only, and non-significant interactions were omitted, until the most parsimonious model was selected. Where significant interactions existed, general linear hypothesis tests using Tukey’s all-pair comparisons were used to determine which combinations differed. While not all comparisons were of interest, this more conservative approach was considered to be appropriate given that multiple models were assessed. Appropriateness of the fit of the final models was assessed using standard residual plots.

For nipple position (N-HSPJ distance) and intra-oral depth, fixed effects of interest were tongue position (tongue up/tongue down) and group (Pain, Control). For nipple diameters, distance from the tip of the nipple (location: 2, 5, 10, and 15 mm) was also included. All variables were treated as factors. In each case, the starting model had the highest order interactions (position * group; position * group * location) included.

## 3. Results

### 3.1. Subjects

There were 25 dyads recruited for the Control group and 25 for the Pain group. The mothers were 33 ± 4 years old and all were married or in a de facto relationship. All had completed high school education and at least two-thirds had a university degree. Fourteen of the Control group were primiparous, 11 of the Pain group were primiparous, with information unavailable for four. Data for infants are shown in Table 1. There were no differences between the participants in the two groups with respect to any characteristics with the exception of the following: bottle feeding (expressed breastmilk and/or infant formula) was introduced significantly earlier in Pain group compared to Control group (*p* < 0.001), and there was a non-significant trend for expressed breast milk to be fed in Pain group (*p* = 0.068).

**Table 1:** Characteristics of infants of Control group (mothers with no nipple pain) and Pain group (mothers experienced persistent nipple pain during breastfeeding).

Characteristic	Control Group *n* = 25	Pain group *n* = 25
Infant		
Age (d)	48 (35, 61)	46 (30, 70)
Sex (Male)	12	12
Caesarean	10/25	6/21
Gestational age (wk)	40 (39, 40)	39 (39, 40)
Birth weight (kg)	3.5 (3.3, 3.7)	3.5 (3.1, 3.8)
Birth length (cm)	51 (50, 53)	51 (49, 52)
First breastfeed (min after birth)	60 (18, 135)	60 (30, 180)
Bottle (yes)	13/25	14/19
Age introduced (d postpartum)	42 (24, 42)	6 (2, 42)
Expressed Breastmilk	11/25	14/19
Formula	4/25	4/19
Pacifier (yes)	15/24	11/20
Age introduced (d postpartum)	20 (7, 25)	14 (5, 23)

Data are presented as mean ± SD, or median (IQR), or proportion; Totals not commensurate with group numbers indicate missing data.

### 3.2. Tongue Movement

**Figure 2:**
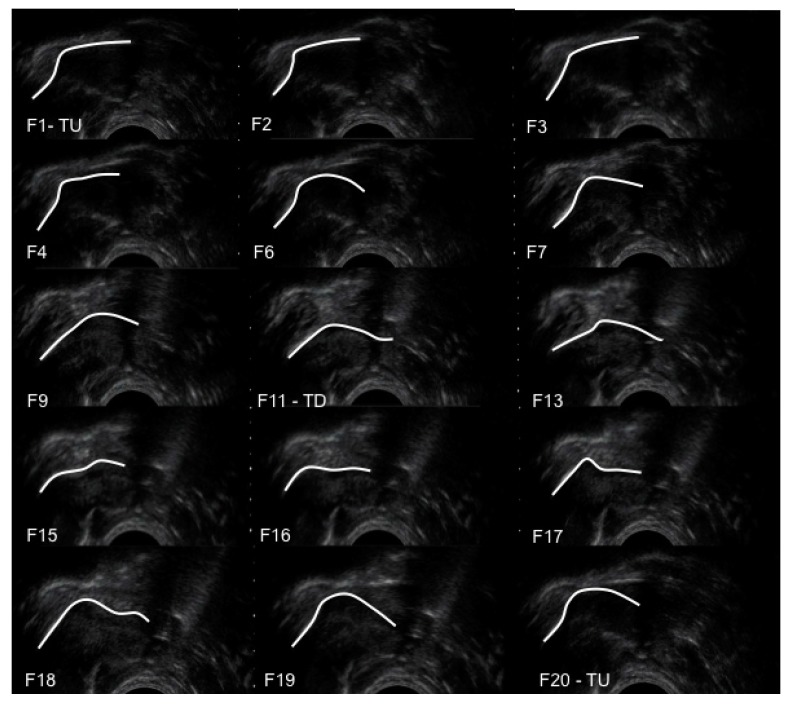
Sequential submental ultrasound frames (F1 to F20) of the infant intra-oral cavity with the tongue contour marked in white. TU, tongue up; TD, tongue down.

The temporal sequence of tongue movement over a suck cycle is shown in an ultrasound image sequence for a control infant in Figure 2. From F1 (tongue up) to F9 the tongue moved inferiorly with the mid-tongue showing most displacement as it moved away from the HSPJ. At F11 (tongue down) the mid-tongue was at its lowest point. From F11 to F20 the mid-tongue rose sequentially in an anterior posterior fashion. The anterior tongue of several infants was seen to rise slightly before the mid-tongue had reached the nadir, but there was no consistent difference between Control group and Pain group.

As the mid-tongue lowered from the HSPJ milk could be seen to fill the intra-oral space bounded distally by the tip of the nipple, proximally by the soft palate (which was in apposition with the posterior tongue), superiorly by the hard palate and inferiorly by the dorsal surface of the tongue. The soft palate tracked the posterior tongue as it moved inferiorly with the mid-tongue, and as the mid-tongue returned to the hard palate milk could be seen to pass under the soft-palate within two frames (<0.08 s).

### 3.3. Nipple Position and Intra-Oral Space Depth 

The distance of the tip of the nipple to the HSPJ (N-HSPJ distance); was not significantly different between Control group and Pain group at either tongue up or tongue down. The decrease in average N-HSPJ distance from tongue up to tongue down was 3.9 mm for Control group (*p* < 0.001) and 4.2 mm for Pain group (*p* < 0.001). The depth of the intra-oral space was not different between Control group and Pain group at tongue up, but it was greater for Control group compared to Pain group when the mid-tongue was maximally lowered (Table 2).

**Table 2:** Nipple position and depth of intra-oral space in the tongue up and the tongue down positions for Control group (mothers with no nipple pain) and Pain group (mothers experienced persistent nipple pain during breastfeeding).

Measurement	Position	Control Group *n* = 25	Pain Group *n* = 25	*p*-value
N-HSPJD (mm)	TU	8.1 ± 0.3 (1.9, 13.9)	8.4 ± 0.6 (3.5, 17.0)	0.977
	TD	4.3 ± 0.4 (0.0, 10.1)	5.1 ± 0.6 (0.5, 10.9)	0.537
Depth (mm)	TU	0.3 ± 0.2 (0.0, 2.7)	0.2 ± 0.1 (0.0, 3.5)	0.963
	TD	7.5 ± 0.2 (4.0, 12.8)	6.7 ± 0.2 (3.5, 14.5)	0.040

Data are presented as mean ± SEM (range) for three suck cycles of each infant; N-HSPJD, nipple to hard soft palate junction distance; Depth, depth of the intra-oral space at the mid tongue; TU, tongue up; TD, tongue down *n* = 75 measurements.

### 3.4. Nipple Shape

**Figure 3:**
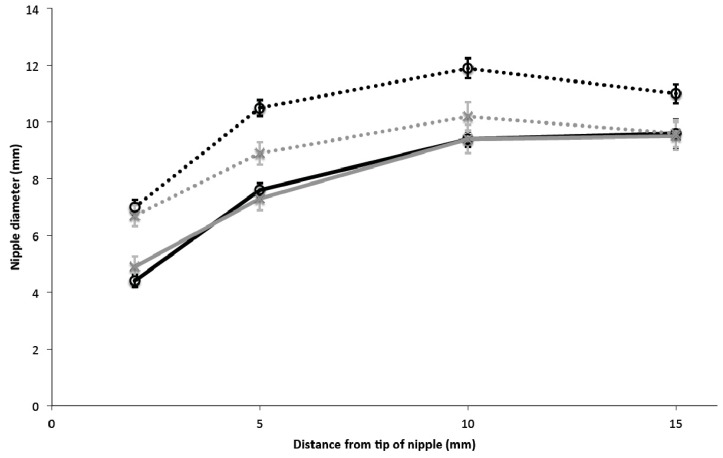
Nipple diameter at 2, 5, 10 and 15 mm from the tip of the nipple in the Control group (black lines) and Pain group (grey lines) in the tongue up position (solid lines) and the tongue down position (dashed lines). Data are presented as mean ± SEM for three suck cycles for each of 25 Control group infants and 25 Pain group infants.

At tongue up there was no difference in nipple diameter between Control group and Pain group at any location (*p >* 0.481, Figure 3). The diameter of the nipple 2 mm from the tip of the nipple was smaller (*p* < 0.001) than at 5 mm from the tip of the nipple, which was smaller (*p* < 0.001) than at 10 mm or 15 mm from the tip of the nipple. There was no difference in diameter between 10 mm and 15 mm from the tip of the nipple (*p* = 1).

At tongue down compared with tongue up the nipple diameter for Control group increased at all locations (*p* < 0.01), but for Pain group, while the nipple diameter increased at 2 mm, 5 mm and 10 mm from the tip of the nipple (*p* < 0.01) there was no significant increase at 15 mm (*p* = 1).

Comparing Pain group and Control group at tongue down, there was no significant difference at 2 mm from the tip of the nipple (*p* = 0.873), but there were differences at 5 mm, 10 mm and 15 mm from the tip of the nipple (*p* < 0.001, *p* = 0.002, *p* = 0.012, respectively) (Figure 3). In Pain group at tongue down, compared to tongue up, between 2 mm and 5 mm from the tip the nipple expanded by 1.6–1.8 mm (*p* < 0.001), and at 10 mm from the tip it expanded by 0.8 mm (*p* = 0.007), while the base of the nipple at 15 mm from the tip remained unchanged (*p* = 1). In Control group at tongue down, compared to tongue up, between 2 mm and 10 mm from the tip the nipple expanded by 2.5–2.8 mm (*p* < 0.001), while the base of the nipple at 15 mm from the tip expanded by 1.4 mm (*p* < 0.001) (Figure 3). The expansion of the nipple between 2 mm and 10 mm from the tip was less for Pain group compared with Control group (*p* = 0.018).

### 3.5. Milk Transfer and Intra-Oral Vacuum

There was no significant difference in milk transfer volume between Pain group and Control group. However, mean milk transfer rate was 3.1 g/min slower for Pain group than for Control group, and milk transfer rate during nutritive sucking was 3.6 g/min slower for Pain group than Control group (Table 3).

**Table 3:** Milk transfer and vacuum characteristics for Control group (mothers with no nipple pain) and Pain group (mothers experienced persistent nipple pain during breastfeeding).

Measurement	Control Group *n* = 25	Pain Group *n* = 25	*p*-Value
Milk transfer volume (g) **^a^**	78.2 ± 34.7 (22)	55.1 ± 38.7 (19)	0.069
Mean milk transfer rate (g/min)	8.9 ± 4.2 (22)	5.8 ± 3.8 (19)	0.024
Nutritive mean milk transfer rate (g/min)	13.7± 5.8 (21)	10.1 ± 4.8 (16)	0.028
Baseline vacuum (mmHg)	−52.5 ± 32.2 (25)	−95.2 ± 61.4 (22)	0.008
Peak vacuum during nutritive sucking (mmHg)	−170.9 ± 68.6 (25)	−237.7 ± 59.6 (22)	0.002

Data are presented as mean ± SD (*n*); *n* not commensurate with group numbers indicate missing data; **^a^** Milk transfer was measured by test weighing where 1 g is equivalent to 1 mL.

Significantly stronger baseline and peak vacuums were measured during nutritive sucking for Pain group compared to Control group (Table 3).

## 4. Discussion

The analysis of ultrasound images in this study has demonstrated that Pain group infants showed reduced tongue movement compared to Control group infants resulting in significantly smaller expansion of the middle of the nipple and no expansion of the base of the nipple. We have confirmed that infants of mothers with nipple pain exert stronger sucking vacuums [10]. Although the milk transfer volume of Pain group in this study was higher than found in the previous study [10] there was still a trend for this to be lower than Control group. The milk transfer rate during nutritive sucking was higher in this Pain group compared to the previous measure of 6.6 g/min (derived from data presented in [10]), yet it was still slower than for infants of mothers who were not experiencing nipple pain.

During nutritive sucking the greatest degree of tongue movement (7.2 mm for Control group) occurred where the mid-tongue lowered from the HSPJ as vacuum within the oral cavity increased and milk flow was observed, filling the intra-oral space. These data are consistent with previous observations [8]. The decreased depth of the intra-oral space at tongue down in Pain group compared with Control group is consistent with the lower milk transfer rate in Pain group. As the tongue moved superiorly milk was cleared under the soft palate and over the surface of the posterior tongue to the pharyngeal area (Figure 2) and vacuum returned to baseline levels. Thus, compression of the nipple in the second half of the suck cycle was associated with oral swallowing, rather than with milk flow.

The relatively even expansion of the nipple of 2.6–2.8 mm between 2 mm and 10 mm from the tip in Control group is consistent with previous results [12,17]. The rise in the anterior tongue slightly before the mid-tongue had reached the nadir in some infants may have contributed to the smaller expansion measured at the base of the nipple. The smaller expansion of the nipple overall, the minimal expansion of 0.8 mm at 10 mm from the tip of the nipple and the lack of any expansion at the base of the nipple in Pain group may be associated with the clinical observation of distorted nipple shapes post-breastfeeding in women with nipple pain. According to the principles of fluid dynamics flow will be restricted by the minimum duct diameter. The tongue movements limiting the expansion of the nipple may have also contributed to the slower milk transfer measured in the Pain group by not allowing milk ducts to fully expand during lowering of the tongue [8]. Thus, correct positioning and attachment of the infant may ensure that the milk ducts just below the areolar surface [18] are within the mouth, and able to expand when the tongue lowers.

Optimal positioning of the nipple is likely to be important for co-ordination of milk removal and swallowing and may also resolve the pain of many mothers. Demarcation of the nipple and areola is not distinguishable on ultrasound images; therefore it was not possible to objectively assess the depth of attachment in this study. However, we found that the nipple was positioned similarly (reflected by the N-HSPJ distance) in both Control group and Pain group infants. It is possible that advice on positioning and attachment given to these mothers by lactation consultants ensured that any cases of shallow attachment had already been corrected.

The baseline vacuum applied by Pain group infants was approximately double that applied by Control group infants, similar to previous results [10]. This strong baseline vacuum may have been applied by the infant in order to achieve and maintain an optimal N-HSPJ distance, and could conceivably contribute to the pain experienced by the mother [6,10]. Baseline vacuum typically varies between infants and this may be related to nipple and breast morphology as well as infant oral anatomy and attachment to the breast [8,19]. Therefore, further studies are required to investigate the impact of nipple and breast morphology and infant attachment to the breast on strength of vacuum and tongue movement during breastfeeding. As baseline vacuum was predictive of the strength of peak vacuum it is important to identify factors conducive to a strong baseline vacuum in order to resolve persistent nipple pain. Ideally, infants’ vacuum levels should be comfortable yet sufficient for attachment and efficient, effective milk removal.

Successful breastfeeding is reliant upon a number of maternal and infant factors, and infant sucking plays a major role through milk removal. The importance of the infant to breastfeeding success is indicated by the abnormal tongue movements associated with reduced feeding efficiency in the Pain group infants. Abnormal tongue movements have also been visualized in infants with ankyloglossia, with some compressing the base of the nipple and others compressing the tip of the nipple, and frenulotomy was shown to normalize the tongue movement, that was associated with improved LATCH score, decreased pain score and improved milk intake [20]. Research on bottle-feeds has shown that infant sucking is adaptable and dependent upon teat rigidity and milk flow rate [21,22,23], but no data exist that demonstrate the breastfeeding infant’s response to variations in breast anatomy, infant positioning, or alternate feeding experiences. Pain group infants were introduced to bottle feeding earlier than Control group infants, which may have been a way of relieving nipple pain, however we cannot determine whether this affected infant sucking development. Maternal factors that could contribute to abnormal infant sucking and milk transfer include smaller/non-protractile nipples and constriction due to vasospasm [24,25]. The Pain group had already received professional advice, and external observation indicated they had achieved optimal positioning and attachment. However, this apparent improvement in positioning and attachment may have been made possible by the abnormal tongue movement and stronger sucking vacuums that were required due to variations in infant and maternal morphology. Thus, simultaneous measurement using the techniques described in this study as well as palate and nipple shape, nipple elasticity and tongue mobility may also be useful, especially during the first 14 days *post partum* when milk production is being established and nipple pain appears to be most prevalent. Development of methods to monitor breastfeeding dyads will aid in determining the success of interventions and rapid resolution of breastfeeding problems.

### Limitations

This study included a small convenience sample of mothers with persistent nipple pain, and the characteristics of their nipples was not assessed. In addition, although infants with ankyloglossia were specifically excluded, no further assessment of infant oral anatomy was undertaken.

## 5. Conclusions

Infants normally use a tongue motion that expands the nipple relatively evenly creating a vacuum to assist in milk removal from the breast. In a population of breastfeeding mothers experiencing persistent nipple pain abnormal infant tongue movements and nipple expansion were observed in association with high intra-oral vacuums and less efficient milk transfer. Measurement of intra-oral vacuum and/or tongue movement via ultrasound may be a useful tool in monitoring treatment strategies in breastfeeding women experiencing pain.
